# Feasibility of Novel Magnetically Controlled Cable Capsule Endoscopy System In Vitro Experiments for Gastric Examination

**DOI:** 10.1155/2022/4313647

**Published:** 2022-10-18

**Authors:** Yaoping Zhang, Lina Qu, Yani Gou, Jinyong Hao, Xiaojun Huang

**Affiliations:** ^1^Department of Gastroenterology, Lanzhou University Second Hospital, Lanzhou, Gansu Province, China; ^2^Gansu Provincial Digestive Endoscopy Engineering Research Center, Lanzhou, Gansu Province, China

## Abstract

**Background:**

Magnetically controlled capsule endoscopy has been shown to be feasible for the examination of gastric diseases. However, there may be problems, such as low image quality, incomplete esophageal observation, and capsule retention. We developed a novel magnetically controlled cable capsule endoscopy (MCCCE) system and evaluated its feasibility through in vitro experiments.

**Methods:**

Three experienced endoscopists performed MCCCE on the plastic stomach model and the excised porcine stomach model 5 times, respectively. Outcomes included handle ability, examination time, examination completion, and image quality. The examination completion was accessed by other two blinded endoscopists, and the image quality was compared with conventional gastroscopy (Olympus, GIF-290).

**Results:**

The performance of MCCCE in vitro experiments is excellent, with a mean operation time of 18.5 minutes in the plastic stomach model and 17.3 minutes in the excised porcine stomach model. The visualization rate of the gastric mucosa is >90% in the plastic stomach model and 75–90% in the excised porcine stomach model. The images of MCCCE are very clear, with good color resolution and no image distortion, which seem to be comparable to conventional gastroscopy.

**Conclusions:**

MCCCE system is feasible for gastric examination in vitro experiments, living animal experiments and human trials need to be further conducted.

## 1. Introduction

Capsule endoscopy has developed by leaps and bounds in the past 20 years, and its indications have also expanded from the initial small intestine to the entire digestive tract today [1]. The concept of magnetically controlled capsule endoscopy (MCCE) was first proposed by Carpi et al. [2] in 2006, which is mainly used for the observation of the gastric disease. At present, there are three main types of MCCE in clinical application: handle type, MRI type, and robotic type [3]. A large number of studies have shown that the diagnostic capability and value of MCCE in gastric diseases are comparable to that of conventional electronic gastroscopy, and it has higher comfort and patient acceptance [4–6].

However, due to the high cost of MCCE and the large equipment, which is inconvenient to move, it has not been widely used especially in primary medical units. Like small intestinal capsule endoscopy, MCCE also has a risk of capsule retention, which limits its application in certain populations, such as patients with intestinal obstruction [7]. Based on the above deficiencies, we developed a novel magnetically controlled cable capsule endoscopy (MCCCE) system. It is the prototype system of wired transmission MCCE system, which was proposed in our another research [8]. In this study, the feasibility of MCCCE in gastric examination was evaluated through in vitro experiments.

## 2. Methods

### 2.1. MCCCE System

**Figure 1:**
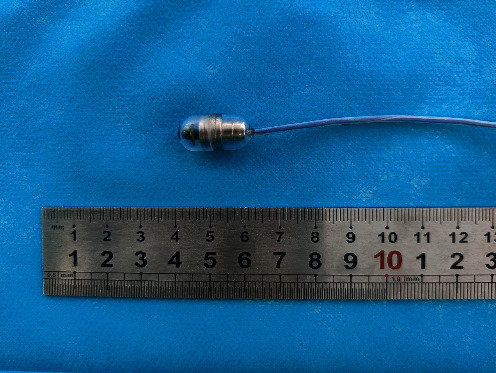
The capsule endoscope and transmission cable.

The MCCCE system consists of a cable capsule endoscope, an imaging device, and a hand-held guidance magnet. The cable capsule endoscope is composed of a capsule endoscope, a transmission cable, and a USB plug (Figure 1).

The capsule endoscope is 11 × 20 mm in size and 3.6 g in weight. It has an internal permanent magnet, six light-emitting diodes (LEDs), and an optical module. The images are transmitted at 30 frames per second (fps), and the image resolution is 1280 × 720 pixels. The transmission cable is 1500 mm in length and 1.8 mm in diameter. The USB plug connects the capsule endoscope to the imaging device, providing power to the capsule endoscope and transmitting the image signal in real time through the transmission cable.

### 2.2. Plastic Stomach Model and Excised Porcine Stomach Model (Figure 2)

#### 2.2.1. Plastic Stomach Model

Three-dimensional modeling: select an adult's standard upper abdominal CT imaging data for three-dimensional modeling. 3D printing: select resin material to 3D print the stomach model according to the built model. Model marking: mark important anatomic sites with numbers in the cavity of the stomach model. Model fixing: hang the marked model in a transparent plastic box to simulate the human abdomen [8].

#### 2.2.2. Excised Porcine Stomach Model

Cleaning: cut the excised porcine stomach from the fundus and turn out the mucosa for washing. Using defoaming agent and mucus removal agents to cope with bubbles, gastric juice and debris in the stomach. Suturing: turn out the serosa and suture the incision of fundus and the distal end of the pylorus. Filling: inject 1000 ml clean water from the cardia and place the porcine stomach in a plastic tray.

### 2.3. Procedure

#### 2.3.1. Plastic Stomach Model

**Figure 3:**
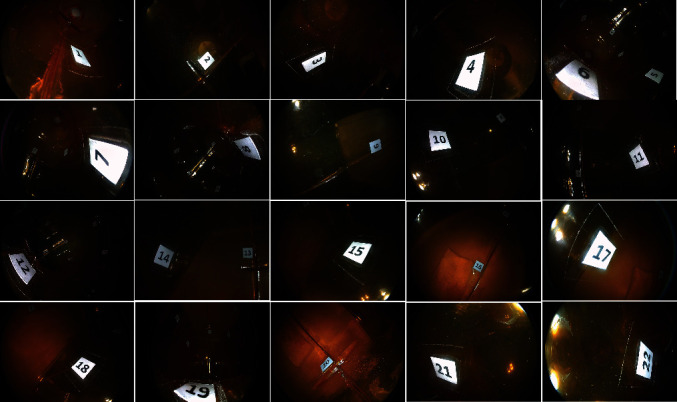
Images taken by MCCCE in the plastic stomach model.

The plastic stomach model was examined with MCCCE by three endoscopists who have experience in performing MCCE. To ensure the complete observation of the entire gastric cavity, at least 22 pictures should be kept according to the “systematic screening protocol for the stomach (SSS)” [9]. With the antegrade view: keep pictures of 4 quadrants of the gastric antrum, body, and middle–upper body. With the retroflex view: keep pictures of 4 quadrants of the gastric antrum, body, and middle–upper body (Figure 3).

#### 2.3.2. Excised Porcine Stomach Model

**Figure 4:**
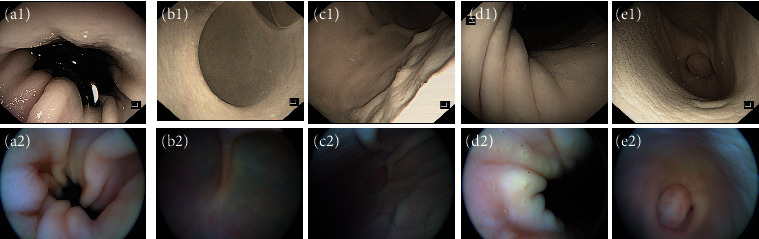
Images taken by conventional gastroscopy and the magnetically controlled cable capsule endoscopy (MCCCE) system in the excised porcine stomach model. (a1–e1): Cardia, fundus, gastric body, gastric angle, antrum, and pylorus detected by conventional gastroscopy. (a2–e2): The same landmarks detected by MCCCE.

The model was examined by the same endoscopists who operated on the plastic stomach model, and the procedure was the same as that of the plastic stomach model. After the MCCCE examination, the model was examined with conventional gastroscopy by other three endoscopists. Then, the pictures taken by the MCCCE and the conventional gastroscopy were compared by two blinded experienced endoscopists to evaluate the quality of the pictures and examination completion (Figure 4).

### 2.4. Main Outcome Measures

Handle ability: excellent (magnetic handle can control the movement of the capsule and precisely reach the target site), good (magnetic handle can control the movement of the capsule but cannot precisely reach the target site), and poor (magnetic handle cannot control the movement of the capsule).

Examination time: complete time of MCCCE from entry to exit from the plastic stomach model and the excised porcine stomach model.

Examination completion: the visualization rate of the gastric mucosa, which is divided into four levels: excellent (>90%), good (75–90%), average (50–75%), and poor (<50%).

Image quality: excellent (the picture is very clear), good (the picture is not very clear but does not affect observation), and poor (the picture is blurry and affects observation).

## 3. Results

Handle ability: the performance of MCCCE in the plastic stomach model and the excised porcine stomach model is excellent, and magnetic handle can control the movement of the capsule and precisely reach the target site.

Examination time: the mean operation time is 18.5 minutes in the plastic stomach model and 17.3 minutes in the excised porcine stomach model.

Examination completion: the visualization rate of the gastric mucosa is >90% in the plastic stomach model and 75–90% in the excised porcine stomach model by the MCCCE. In addition, all marked landmarks in the plastic stomach model are clearly visible.

Image quality: the images of MCCCE are defined excellent, which are very clear, with good color resolution and no image distortion.

## 4. Discussion

Capsule endoscopy has been used in the diagnosis of small intestinal diseases since its introduction in 2000 [10]. In recent years, the emergence of MCCE has explored a new mode for the diagnosis of upper gastrointestinal diseases [11]. Although studies have shown that MCCE is more comfortable than conventional gastroscopy and has the comparable diagnostic ability as gastroscopy, it is not widely used because of the high cost, large equipment, and inconvenient movement. At present, conventional gastroscopy is still the gold standard for the diagnosis of upper gastrointestinal diseases. However, the painful process of gastroscopy may cause many patients who are necessary for gastroscopy to refuse the examination. Although painless gastroscopy can improve comfort, it increases costs and risks due to the use of anesthetics [12, 13]. Based on the advantages and disadvantages of MCCE and conventional gastroscopy, we proposed a novel MCCCE system. This study is a pilot study to evaluate the feasibility of MCCCE system for gastric examination in vitro experiments.

The novel MCCCE system has the following advantages: (1) the capsule endoscope is smaller, easy to swallow, more comfortable, and unnecessary for anesthesia. (2) Capsule endoscope adopts wired transmission to ensure power supply and high-quality image. (3) Capsule endoscope is disposable, and there is no need for post-processing and no risk of cross-infection. (4) Capsule endoscope can be removed by mouth after the examination, and there is no risk of capsule retention. (5) The whole operating system is small, low cost, portable, and easy to operate, which can be used in places where conventional gastrointestinal endoscopy cannot be used, such as hospital bedside and primary medical units.

In this study, three endoscopists who have experience in operating MCCE participated in the experimental operation, and both of them thought MCCCE has excellent performance in the plastic stomach model and the excised porcine stomach model. The capsule can reach any part inside of the plastic stomach model and the excised porcine stomach model under the control of the magnetic handle to achieve a complete observation of the gastric gravity. Each endoscopist repeated the examination 5 times in the plastic stomach model and the excised porcine stomach model with a mean operation time of 18.5 and 17.3 minutes, respectively.

After examination, two blinded experienced endoscopists reviewed pictures taken by MCCCE and accessed the examination completion. The visualization rate of the gastric mucosa is >90% in the plastic stomach model and 75–90% in the excised porcine stomach model, and all marked landmarks in the plastic stomach model can be clearly observed. By comparing with the images of conventional gastroscopy, MCCCE's images taken in the excised porcine stomach model are as clear as conventional gastroscopy, with good color resolution and no image distortion, which can accurately assess the condition in the gastric cavity and the mucosal surface. It shows that the MCCCE can meet the clinical requirements for upper gastrointestinal endoscopy images.

Although MCCCE seems to be effective in vitro experiments, there are still some limitations of our study. First, the cable of MCCCE may cause some discomfort during examination, such as gag reflex, sore throat, and foreign body sensation. Second, the performance of MCCCE was only tested in the plastic stomach model and the excised porcine stomach model, which do not include esophagus and duodenum. Although planned, the human test has not finished yet. However, it is worth mentioning that a research by Chen et al. [14] proposed a detachable string magnetically controlled capsule endoscopy (DS-MCE). This pilot study involved 4 healthy volunteers and 21 patients with suspected esophageal disease to assess the feasibility and safety of DS-MCE. The results showed that DS-MCE was safe, well-tolerated, and feasible for viewing the esophagus. Since the design of MCCCE is similar to DS-MCE, it can be inferred that MCCCE will also have good tolerability and feasibility for upper gastrointestinal examination in human.

In conclusion, the novel MCCCE system is feasible for gastric examination in vitro models, living animal experiments and human trials are necessary to evaluate its safety, tolerability, and diagnostic accuracy.

## Figures and Tables

**Figure 2:**
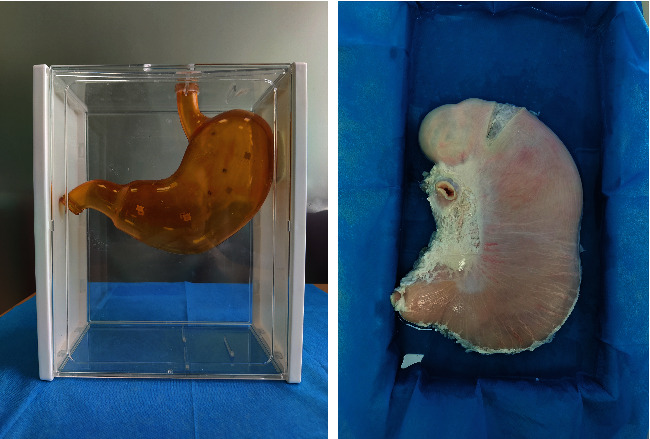
Plastic stomach model and excised porcine stomach model.

## Data Availability

Data sharing is not applicable to this article as no new data were generated or analysed during the current study.

## Abbreviations

MCCCE: Magnetically controlled cable capsule endoscopy
MCCE: Magnetically controlled capsule endoscopy
LEDs: Light- emitting diodes
fps: Frames per second
SSS: Systematic screening protocol for the stomach
DS-MCE: Detachable string magnetically controlled capsule endoscopy.

## References

[1] Hosoe N., Naganuma M., Ogata H. (2015). Current status of capsule endoscopy through a whole digestive tract. *Digestive Endoscopy*.

[2] Carpi F., Galbiati S., Carpi A. (2006). Magnetic shells for gastrointestinal endoscopic capsules as a means to control their motion. *Biomedicine & Pharmacotherapy*.

[3] Liao Z., Zou W., Li Z. S. (2018). Clinical application of magnetically controlled capsule gastroscopy in gastric disease diagnosis: recent advances. *Science China Life Sciences*.

[4] Liao Z., Hon X., Lin-Hu E. Q., Sheng J. Q., Ge Z. Z., Jiang B. (2017). Accuracy of magnetically controlled capsule endoscopy, compared with conventional gastroscopy, in detection of gastric diseases. *Gastroenterological Endoscopy*.

[5] Qian Y. Y., Zhu S. G., Hou X. (2018). Preliminary study of magnetically controlled capsule gastroscopy for diagnosing superficial gastric neoplasia. *Digestive and Liver Disease*.

[6] Zou W. B., Hou X. H., Xin L. (2015). Magnetic-controlled capsule endoscopy vs. gastroscopy for gastric diseases: a two-center self-controlled comparative trial. *Endoscopy*.

[7] Cortegoso Valdivia P., Skonieczna-Zydecka K., Elosua A. (2022). Indications, detection, completion and retention rates of capsule endoscopy in two decades of use: a systematic review and meta-analysis. *Diagnostics*.

[8] Zhang Y., Qu L., Gou Y., Hao J., Pan Y., Huang X. (2022). In vitro and in vivo evaluation of a novel wired transmission magnetically controlled capsule endoscopy system for upper gastrointestinal examination. *Surgical Endoscopy*.

[9] Yao K. (2013). The endoscopic diagnosis of early gastric cancer. *Annals of Gastroenterology*.

[10] Iddan G., Meron G., Glukhovsky A., Swain P. (2000). Wireless capsule endoscopy. *Nature*.

[11] Rauya E., Sha O., Darwazeh R., Zhang B. Q. (2019). Efficacy and safety of magnetic guided capsule gastroscopy in gastric diseases. *Acta Gastro-Enterologica Belgica*.

[12] Inadomi J. M., Gunnarsson C. L., Rizzo J. A., Fang H. (2010). Projected increased growth rate of anesthesia professional-delivered sedation for colonoscopy and EGD in the United States: 2009 to 2015. *Gastrointestinal Endoscopy*.

[13] Ciriza C., García L., Fernández A., Díez A., Delgado M., San Sebastián A. I. (2001). Sedation for gastrointestinal endoscopy. Analysis of tolerance and complications. *Revista Española de Enfermedades Digestivas*.

[14] Chen Y. Z., Pan J., Luo Y. Y. (2019). Detachable string magnetically controlled capsule endoscopy for complete viewing of the esophagus and stomach. *Endoscopy*.

